# Development of a stable semi-continuous lipid production system of an oleaginous *Chlamydomonas* sp. mutant using multi-omics profiling

**DOI:** 10.1186/s13068-022-02196-w

**Published:** 2022-09-16

**Authors:** Tomoki Oyama, Yuichi Kato, Ryota Hidese, Mami Matsuda, Minenosuke Matsutani, Satoru Watanabe, Akihiko Kondo, Tomohisa Hasunuma

**Affiliations:** 1 Graduate School of Science, Technology and Innovation, Kobe University, 1-1 Rokkodai, Nada, Kobe 657-8501 Japan; 2 Engineering Biology Research Center, Kobe University, 1-1 Rokkodai, Nada, Kobe 657-8501 Japan; 3 NODAI Genome Research Center, Tokyo University of Agriculture, 1-1-1 Sakuragaoka, Setagaya, Tokyo 156-8502 Japan; 4 Department of Bioscience, Tokyo University of Agriculture, 1-1-1 Sakuragaoka, Setagaya, Tokyo 156-8502 Japan; 5 Department of Chemical Science and Engineering, Graduate School of Engineering, Kobe University, 1-1 Rokkodai, Nada, Kobe 657-8501 Japan

**Keywords:** *Chlamydomonas*, Lipid, Semi-continuous culture, Nitrate-replete condition, Nitrogen starvation response

## Abstract

**Background:**

Microalgal lipid production has attracted global attention in next-generation biofuel research. Nitrogen starvation, which drastically suppresses cell growth, is a common and strong trigger for lipid accumulation in microalgae. We previously developed a mutant *Chlamydomonas* sp. KAC1801, which can accumulate lipids irrespective of the presence or absence of nitrates. This study aimed to develop a feasible strategy for stable and continuous lipid production through semi-continuous culture of KAC1801.

**Results:**

KAC1801 continuously accumulated > 20% lipid throughout the subculture (five generations) when inoculated with a dry cell weight of 0.8–0.9 g L^−1^ and cultured in a medium containing 18.7 mM nitrate, whereas the parent strain KOR1 accumulated only 9% lipid. Under these conditions, KAC1801 continuously produced biomass and consumed nitrates. Lipid productivity of 116.9 mg L^−1^ day^−1^ was achieved by semi-continuous cultivation of KAC1801, which was 2.3-fold higher than that of KOR1 (50.5 mg L^−1^ day^−1^). Metabolome and transcriptome analyses revealed a depression in photosynthesis and activation of nitrogen assimilation in KAC1801, which are the typical phenotypes of microalgae under nitrogen starvation.

**Conclusions:**

By optimizing nitrate supply and cell density, a one-step cultivation system for *Chlamydomonas* sp. KAC1801 under nitrate-replete conditions was successfully developed. KAC1801 achieved a lipid productivity comparable to previously reported levels under nitrogen-limiting conditions. In the culture system of this study, metabolome and transcriptome analyses revealed a nitrogen starvation-like response in KAC1801.

**Supplementary Information:**

The online version contains supplementary material available at 10.1186/s13068-022-02196-w.

## Background

Microalgae have attracted global attention as next-generation biofuel producers [1] because of their potential for photosynthetically producing biofuel feedstocks, such as triacylglycerols (lipids), from atmospheric carbon dioxide (CO_2_) [2]. Several microalgal strains, from genera including *Chlorella*, *Nannochloropsis*, *Scenedesmus*, and *Chlamydomonas*, can accumulate lipids at over 50% of dry cell weight (DCW) [3–6]. Lipid production in microalgae is affected by environmental factors, such as light [7, 8], salinity [9, 10], nutrients [11, 12], and temperature [13, 14]. Among them, nitrogen starvation is a common and strong trigger for lipid accumulation [15, 16], while it also drastically suppresses cell growth [17]. Therefore, conventional microalgal cultivation for lipid production is divided into two steps: a nitrogen-replete step for cell growth and a nitrogen-deficient step for lipid accumulation. Semi-continuous culture is performed by periodically harvesting a specific volume of culture broth and adding an equal amount of fresh medium to achieve the desired cell density [18–20]. This method is advantageous for maintaining cell growth, though its utilization for lipid production is limited because of trade-offs between cell growth and lipid accumulation [17].

To realize one-step lipid production, microalgal strains that can accumulate lipids during the cell growth phase have been developed. Ajjawi et al. (2017) identified a transcription factor containing the Zn(II)_2_Cys_6_ binuclear cluster domain in *Nannochloropsis gaditana* and showed that downregulation of the factor caused a twofold increase in lipid production [21]. Fukuda et al. (2018) found that the glycerol 3-phosphate (G3P) acyltransferase gene *GPAT1* enhanced lipid production in *Cyanidioschyzon merolae*: a *GPAT1*-overexpressing strain exhibited 56.1-fold higher lipid productivity than the parental strain [22]. Südfeld et al. showed that a knockout strain of transcription factor NO06G03670 in *Nannochloropsis oceanica* had 1.4-fold higher lipid accumulation than the parental strain [23]. Using a random mutagenesis approach, Oyama et al. developed a *Chlamydomonas* sp. mutant, KAC1801, that accumulated lipids even under nitrate-replete conditions [24]. Thus, while microalgal lipid production with concurrent cell growth has been improved by strain development, cultivation strategies have not been adequately investigated.

This study aimed to achieve stable and continuous lipid production under nitrogen-replete conditions suitable for the growth of *Chlamydomonas* sp. KAC1801. A semi-continuous culture of KAC1801 was performed with abundant nitrate supplementation, and lipid production was compared to that in the parental strain KOR1, which accumulates fewer lipids in the presence of a nitrogen source [25]. Comparative metabolome and transcriptome analyses were also conducted. According to the results, *Chlamydomonas* sp. KAC1801 showed stable lipid productivity rates comparable to previously reported levels in nitrogen-limiting culture systems.

## Results

### Development of a lipid production method by the semi-continuous culture of KAC1801

This study aimed to develop a semi-continuous culture method for KAC1801 to achieve lipid production at levels feasible for commercialization. Light and nitrogen availability are important factors affecting cell growth and lipid accumulation in microalgae [7, 8, 15, 16]. The impact of inoculation cell density and nitrate concentration during semi-continuous cultivation was examined. KAC1801 and the parental strain, KOR1, were subcultured every 24 h at an initial cell concentration of optical density at 750 nm (OD_750_) = 0.5 (0.4–0.5 g L^−1^) or 1.0 (0.8–0.9 g L^−1^) in modified Bold’s (MB) medium containing 9.3 mM (6 N) or 18.7 mM (12 N) NaNO_3_ as the sole nitrogen source. Consistent with a previous report [24], biomass production and nitrate consumption by KAC1801 were lower, whereas lipid production was higher than that in KOR1 (Additional file 1: Fig. S1, Table S1). The mean lipid production in KAC1801 after 5 d of cultivation was 67.0 mg L^–1^ (6 N, OD_750_ = 0.5), 84.0 mg L^–1^ (6 N, OD_750_ = 1.0), 66.6 mg L^–1^ (12 N, OD_750_ = 0.5), and 138.4 mg L^–1^ (12 N, OD_750_ = 1.0), whereas that in KOR1 was 44.4 mg L^–1^ (6 N, OD_750_ = 0.5), 42.9 mg L^–1^ (6 N, OD_750_ = 1.0), 36.9 mg L^–1^ (12 N, OD_750_ = 0.5), and 45.3 mg L^–1^ (12 N, OD_750_ = 1.0) (Additional file 1: Fig. S2, Table S1). Thus, maximal lipid production in KAC1801 was achieved in medium containing 12 N NaNO_3_ at an initial cell density inoculation of OD_750_ = 1.0, suggesting that these conditions are suitable for semi-continuous cultivation of KAC1801.

**Fig. 1:**
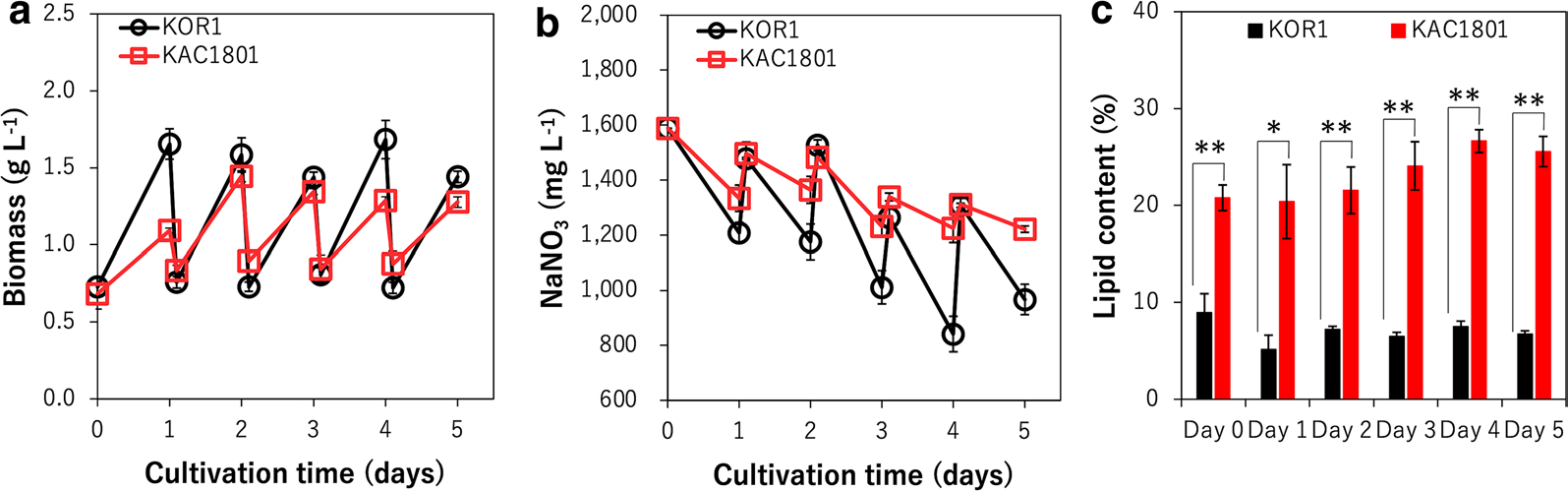
Time-course profiles of *Chlamydomonas* sp. properties during semi-continuous cultivation. **a** Biomass concentration (g L^–1^). **b** Nitrate concentration in the medium (mM). **c** Lipid content (%). Error bars indicate the standard deviation of three replicate experiments (*p < 0.05, **p < 0.01 via Welch’s *t*-test)

To evaluate the rate of lipid production in further detail, the semi-continuous culture of KAC1801 was performed for 5 d in MB 12 N medium by subculturing cells every 24 h at an inoculation cell density of OD_750_ = 1.0 (Fig. 1). The values for biomass production, nitrate consumption, and lipid content during semi-continuous cultivation are summarized in Table 1. Biomass production in KAC1801 varied from 400.1 mg L^–1^ (day 4–day 5) to 610.6 mg L^–1^ (day 1–day 2), and that in KOR1 from 709.2 mg L^–1^ (day 2–day 3) to 925.1 mg L^–1^ (day 0–day 1). Nitrate consumption by KAC1801 varied from 1.1 mM (day 4–day 5) to 3.0 mM (day 2–day 3), and that in KOR1 from 3.6 mM (day 1–day 2) to 6.1 mM (day 2–day 3). The lipid content in KAC1801 was significantly greater than that in KOR1 during the entire cultivation period; KAC1801 constantly accumulated > 20% lipids, whereas the lipid content in KOR1 was < 9% throughout the cultivation period (Fig. 1c). Mean lipid production in KAC1801 was 116.9 mg L^–1^ day^−1^, 2.3-fold greater than that in KOR1 (50.5 mg L^–1^ day^−1^).

**Table 1:** Summary of the semi-continuous culture

Strain	Culture period	Biomass production (mg L^–1^)	Nitrate consumption (mM)	Lipid production (mg L^–1^)
KOR1	Day 0–Day 1	925.1 ± 153.6	4.5 ± 0.4	20.2 ± 15.4
	Day 1–Day 2	825.0 ± 139.3	3.6 ± 0.7	75.1 ± 23.2
	Day 2–Day 3	709.2 ± 62.7	6.1 ± 0.7	40.5 ± 6.7
	Day 3–Day 4	875.7 ± 115.5	5.0 ± 0.5	74.0 ± 5.0
	Day 4–Day 5	717.9 ± 19.1	4.0 ± 0.6	42.5 ± 6.4
	Mean	810.6 ± 95.4	4.6 ± 1.0	50.5 ± 23.7
KAC1801	Day 0–Day 1	408.4 ± 111.8	2.9 ± 0.5	80.7 ± 22.6
	Day 1–Day 2	610.6 ± 58.9	1.6 ± 0.3	140.4 ± 19.4
	Day 2–Day 3	448.5 ± 80.3	3.0 ± 0.2	129.9 ± 56.0
	Day 3–Day 4	442.6 ± 77.4	1.3 ± 0.8	140.3 ± 34.1
	Day 4–Day 5	400.1 ± 50.0	1.1 ± 0.2	92.9 ± 24.3
	Mean	462.0 ± 21.3	2.0 ± 0.9	116.9 ± 28.1

### Distribution of carbon in carbohydrates, proteins, and pigments

**Fig. 2:**
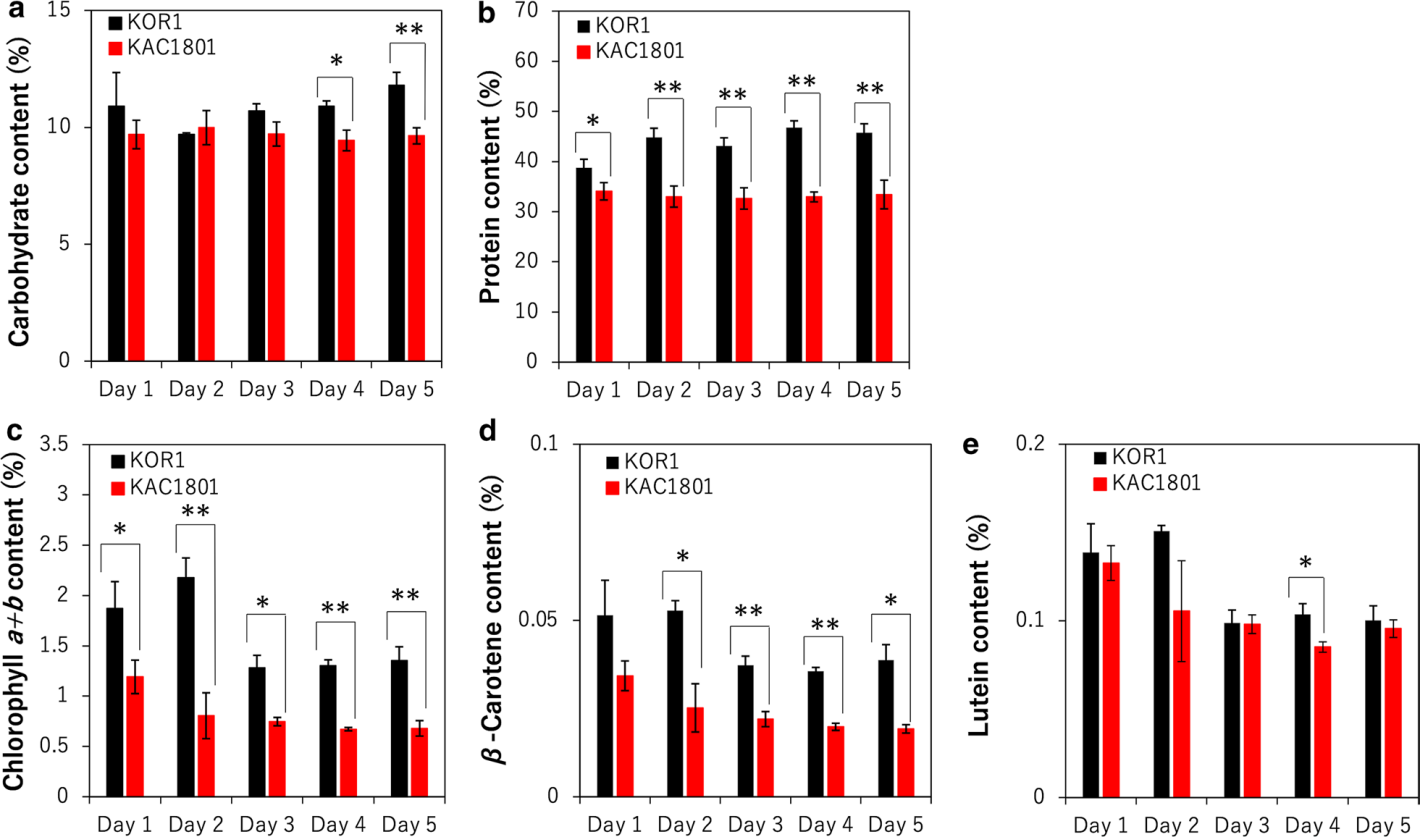
Content of cellular components in *Chlamydomonas* sp. during semi-continuous cultivation. **a** Carbohydrate (%). **b** Protein (%). **c** Chlorophyll *a* + *b* (%). **d** β-Carotene (%). **e** Lutein (%). Error bars indicate the standard deviation of three replicate experiments (*p < 0.05, **p < 0.01 via Welch’s *t*-test)

KAC1801 accumulated more lipids than KOR1 during semi-continuous culture, suggesting an altered carbon distribution resulting from CO_2_ fixation. The levels of other major cell components in the microalga, including carbohydrates, proteins, and photosynthetic pigments, were also analyzed. In KAC1801, carbohydrate content, which is one of the major carbon storage forms in the model species of this study [6, 25], was similar or slightly reduced relative to that in KOR1 (Fig. 2a). The protein and chlorophyll contents in KAC1801 were lower than that in KOR1 throughout the cultivation period (Fig. 2b, c). β-Carotene and lutein are the major carotenoids in the model species of this study [25]; the β-carotene content of KAC1801 was lower than that in KOR1 at almost all sampling points (Fig. 2d). In contrast, the lutein content of KAC1801 was similar or slightly lower than that in KOR1 (Fig. 2e). Thus, in contrast to the results for lipid accumulation, carbon distribution in proteins and photosynthetic pigments was lower in KAC1801 than in KOR1.

### Identification of key metabolic changes involved in the altered carbon distribution in KAC1801

**Fig. 3:**
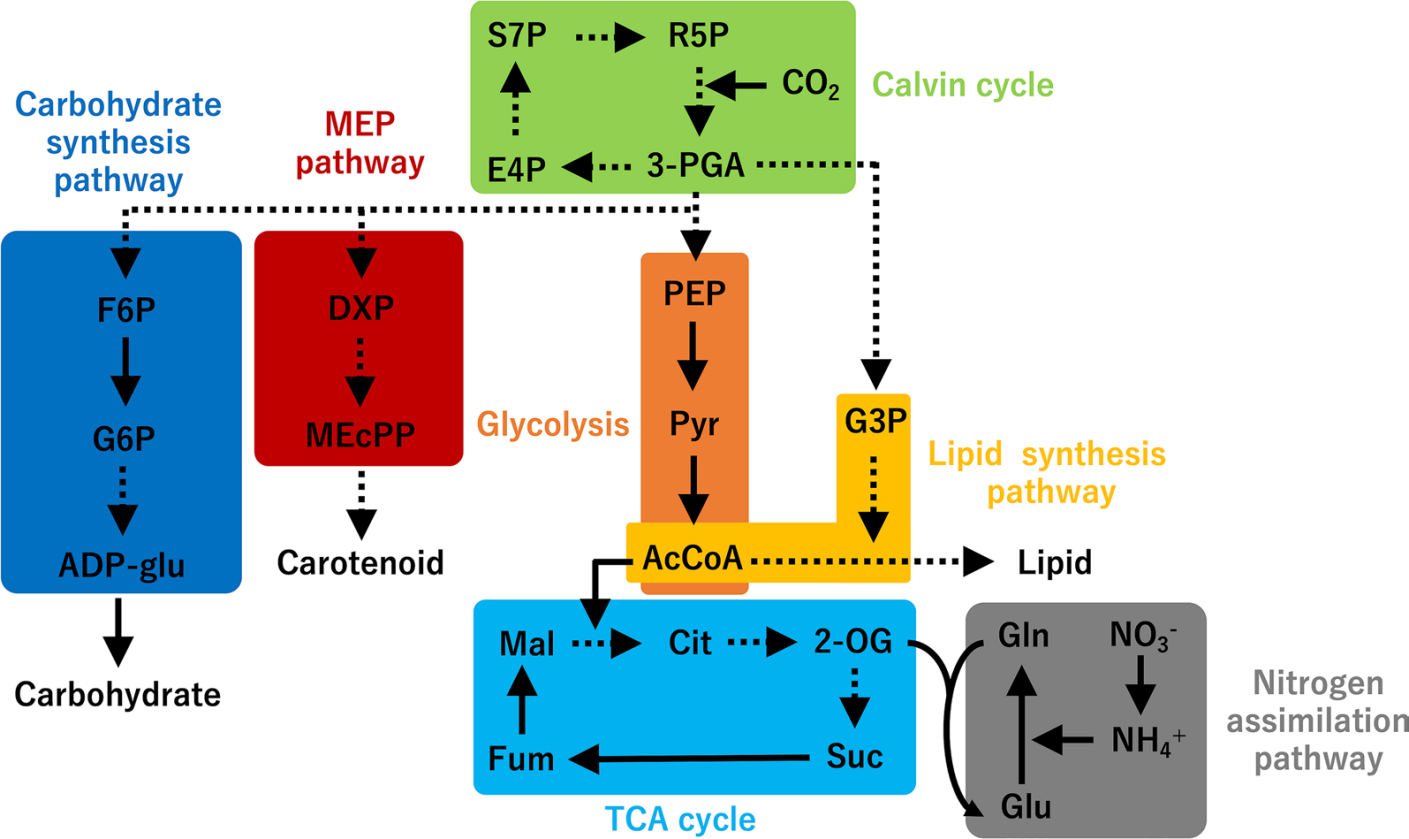
Intracellular metabolites analyzed in this study. Solid and dotted lines represent single and multiple enzymatic steps, respectively. S7P, sedoheptulose 7-phosphate; R5P, ribose 5-phosphate; 3-PGA, 3-phosphoglycerate; E4P, erythrose 4-phosphate; F6P, fructose 6‐phosphate; G6P, glucose 6‐phosphate; ADP-glu, ADP-glucose; DXP, 1-deoxy-d-xylulose 5-phosphate; MEcPP, 2-*C*-methyl-d-erythritol-2,4-cyclopyrophosphate; PEP, phosphoenolpyruvate; Pyr, pyruvate; AcCoA, acetyl-CoA. G3P, glycerol 3-phosphate; Cit, citrate; 2-OG, 2-oxoglutarate; Suc, succinate; Fum, fumarate; Mal, malate; Gln, glutamine; Glu, glutamate; MEP pathway, 2-*C*-methylerythritol 4-phosphate pathway; TCA cycle, tricarboxylic acid cycle

To identify the key metabolites in the altered carbon distribution phenotype, the metabolite pool size in KAC1801 from day 1 to 2 in semi-continuous culture was analyzed by capillary electrophoresis time-of-flight mass spectrometry (CE-TOFMS), according to the Calvin cycle, carbohydrate synthesis pathway, 2-*C*-methylerythritol 4-phosphate (MEP) pathway, glycolysis, lipid synthesis pathway, tricarboxylic acid (TCA) cycle, and nitrogen assimilation pathway (Fig. 3).

**Fig. 4:**
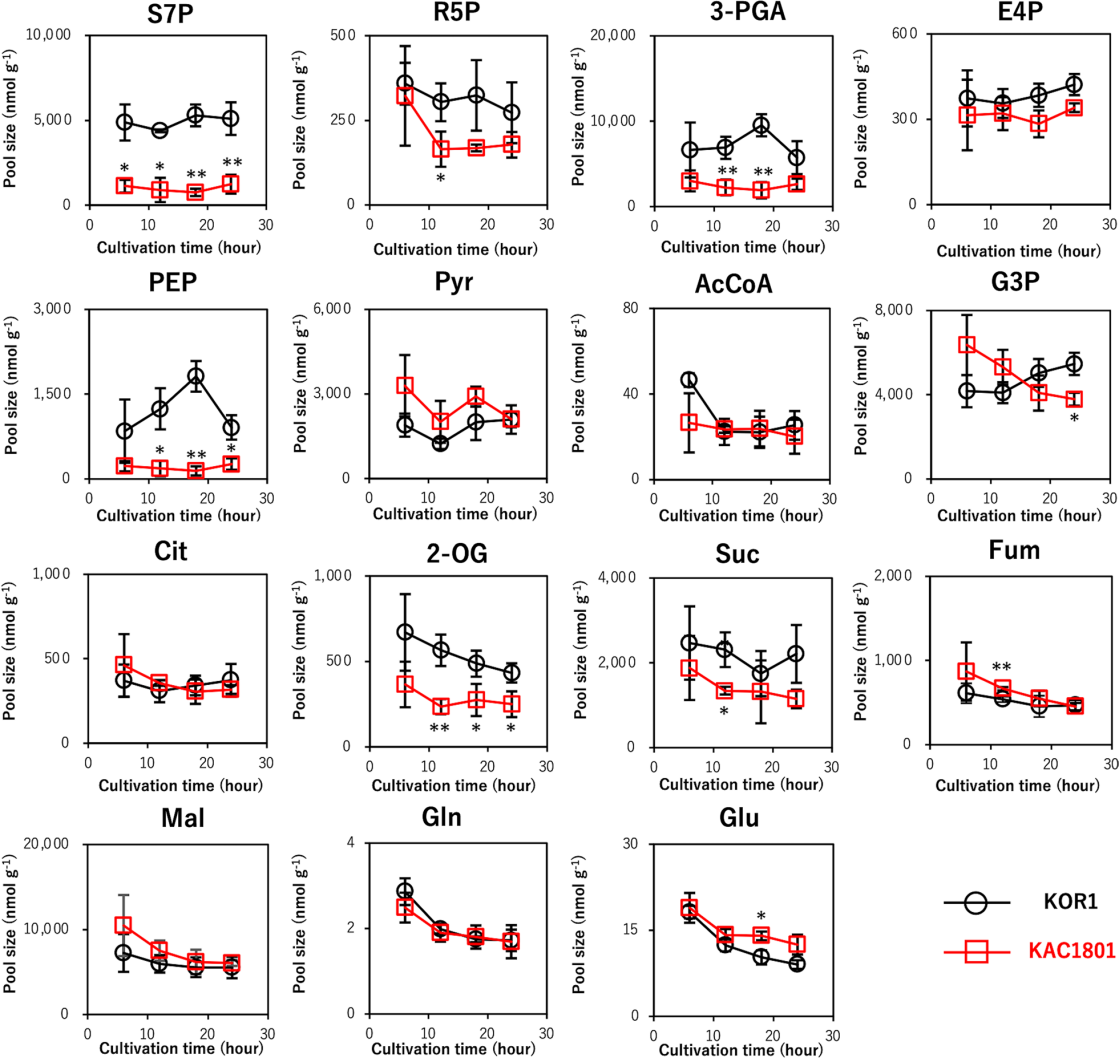
Intracellular metabolite pool size of *Chlamydomonas* sp. during semi-continuous cultivation. KOR1 and KAC1801 cells were subjected to metabolome analysis from day 1 to 2 in semi-continuous culture. Error bars indicate the standard deviation of three replicate experiments (*p < 0.05, **p < 0.01 via Welch’s *t*-test)

In KAC1801, the pool sizes of sedoheptulose 7-phosphate (S7P) and 3-phosphoglycerate (3-PGA) were significantly lower in KAC1801 than in KOR1, while the differences in ribose 5-phosphate (R5P) and erythrose 4-phosphate (E4P) levels were not statistically significant (Fig. 4). These results are consistent with the low photosynthetic pigment content in KAC1801 (Fig. 2) and suggest lower levels of carbon fixation in KAC1801 than in KOR1. During glycolysis, the pool size of phosphoenolpyruvate (PEP) in KAC1801 was significantly lower than that in KOR1, while that of pyruvate (Pyr) and acetyl-CoA (AcCoA) were not different between these strains (Fig. 4). The levels of metabolites in the carbohydrate synthesis pathway, including fructose 6‐phosphate (F6P), glucose 6‐phosphate (G6P), and ADP-glucose (ADP-glu), were similar between KOR1 and KAC1801 (Additional file 1: Fig. S3a–c), consistent with the results for carbohydrate content (Fig. 2a). In microalgae, carotenoid precursors (i.e., isopentenyl pyrophosphate and dimethylallyl diphosphate) are synthesized via the MEP pathway [26]. The pool sizes of the metabolites in the MEP pathway, including 1-deoxy-d-xylulose 5-phosphate (DXP) and 2-*C*-methyl-d-erythritol-2,4-cyclopyrophosphate (MEcPP), were significantly lower in KAC1801 than in KOR1 (Additional file 1: Fig. S3d, e), suggesting a decrease in carbon flux for carotenoid synthesis in KAC1801. This result is consistent with the reduced β-carotene content in KAC1801 (Fig. 2d). In the TCA cycle, the pool sizes of citrate (Cit), malate (Mal), and fumarate (Fum) did not change in KAC1801 over time, except for a significant increase in Fum levels at 12 h (Fig. 4). In contrast, the pool sizes of 2-oxoglutarate (2-OG) and succinate (Suc) were lower in KAC1801 than in KOR1. In nitrogen assimilation, NO_3_^−^ is first converted to NH_4_^+^ by nitrate reductase (NR) and nitrite reductase (NiR) [27]. In the glutamine synthase–glutamate synthase (GS/GOGAT) cycle, glutamine (Gln) is synthesized by glutamine synthase (GS) using NH_4_^+^ and glutamate (Glu) as substrates, and Gln and 2-OG are converted to Glu by glutamate synthase (GOGAT) [28]. The pool size of Glu in KAC1801 was significantly greater than that in KOR1 (Fig. 4).

### ^13^C-turnover analysis of the dynamic metabolic flux in CO_2_ fixation

**Fig. 5:**
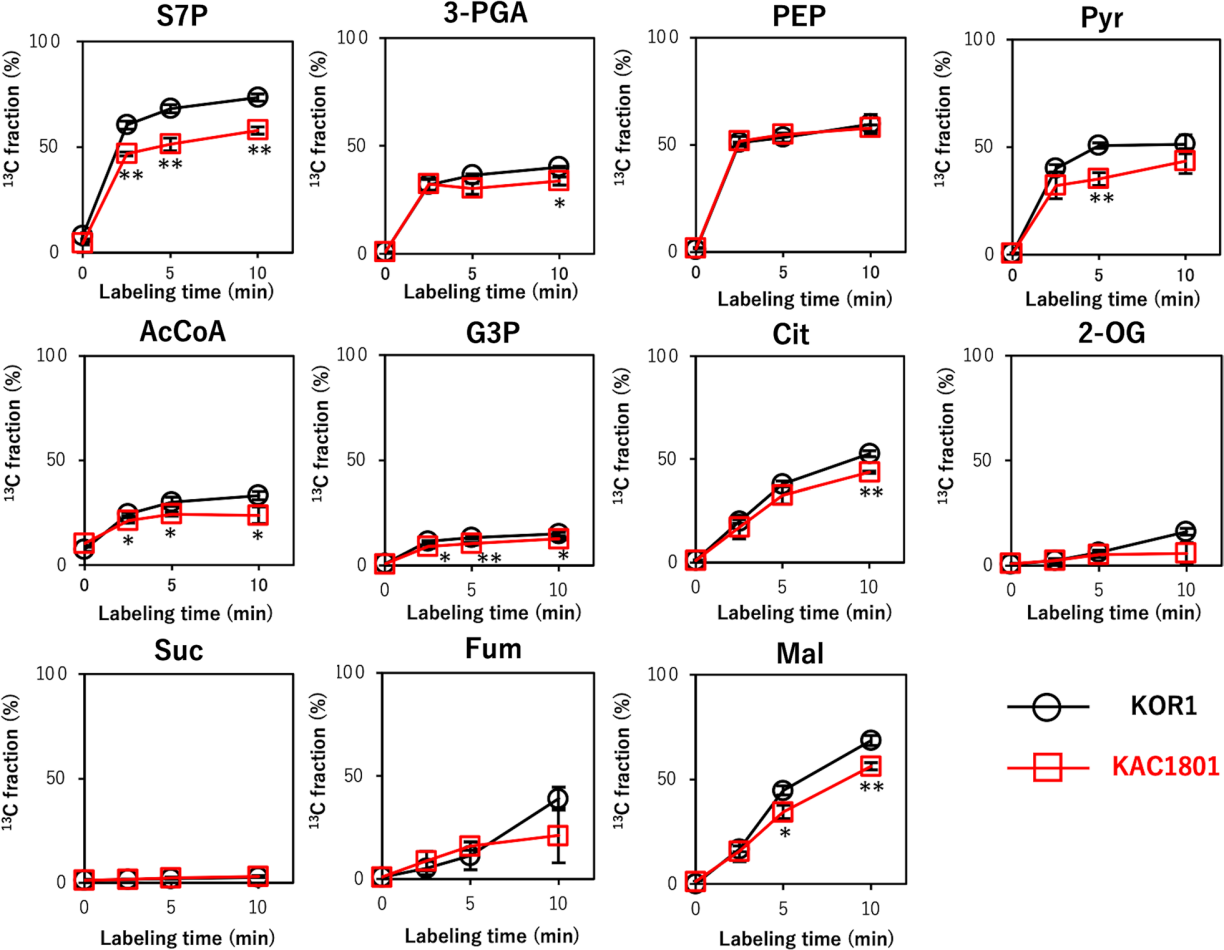
Dynamic metabolic profiling of *Chlamydomonas* sp. during semi-continuous cultivation. KOR1 and KAC1801 cells were harvested on day 1.5 of semi-continuous culture, and intracellular metabolites were labeled with ^13^C by adding NaH^13^CO_3_. The *x*-axes show the labeling time and the *y*-axes show the ^13^C labeling ratio. Error bars indicate the standard deviation of three replicate experiments (*p < 0.05, **p < 0.01 via Welch’s *t*-test)

Analysis of the dynamic changes in metabolite levels is important to understand the metabolic mechanism of the altered carbon distribution [25]. To evaluate metabolic profiles dynamically, the in vivo ^13^C labeling of intracellular metabolites, newly synthesized from radiolabeled-CO_2_, was examined on day 1.5 over 10 min using cells in semi-continuous culture (Fig. 5). In the Calvin cycle, the ^13^C fraction of S7P and 3-PGA of KAC1801 was lower than that in KOR1, suggesting that CO_2_ fixation was decreased in KAC1801. In glycolysis, which occurs downstream of the Calvin cycle, there was no difference in the ^13^C fraction of PEP, and that of Pyr was lower in KAC1801 than in KOR1. The ^13^C fraction of metabolites related to lipid synthesis, including AcCoA and G3P, was lower in KAC1801 than in KOR1. In the TCA cycle, the ^13^C fraction of Cit and Mal was lower in KAC1801, while that of 2-OG, Suc, and Fum was similar in these strains.

### Identification of key genes differently expressed in KAC1801

**Fig. 6:**
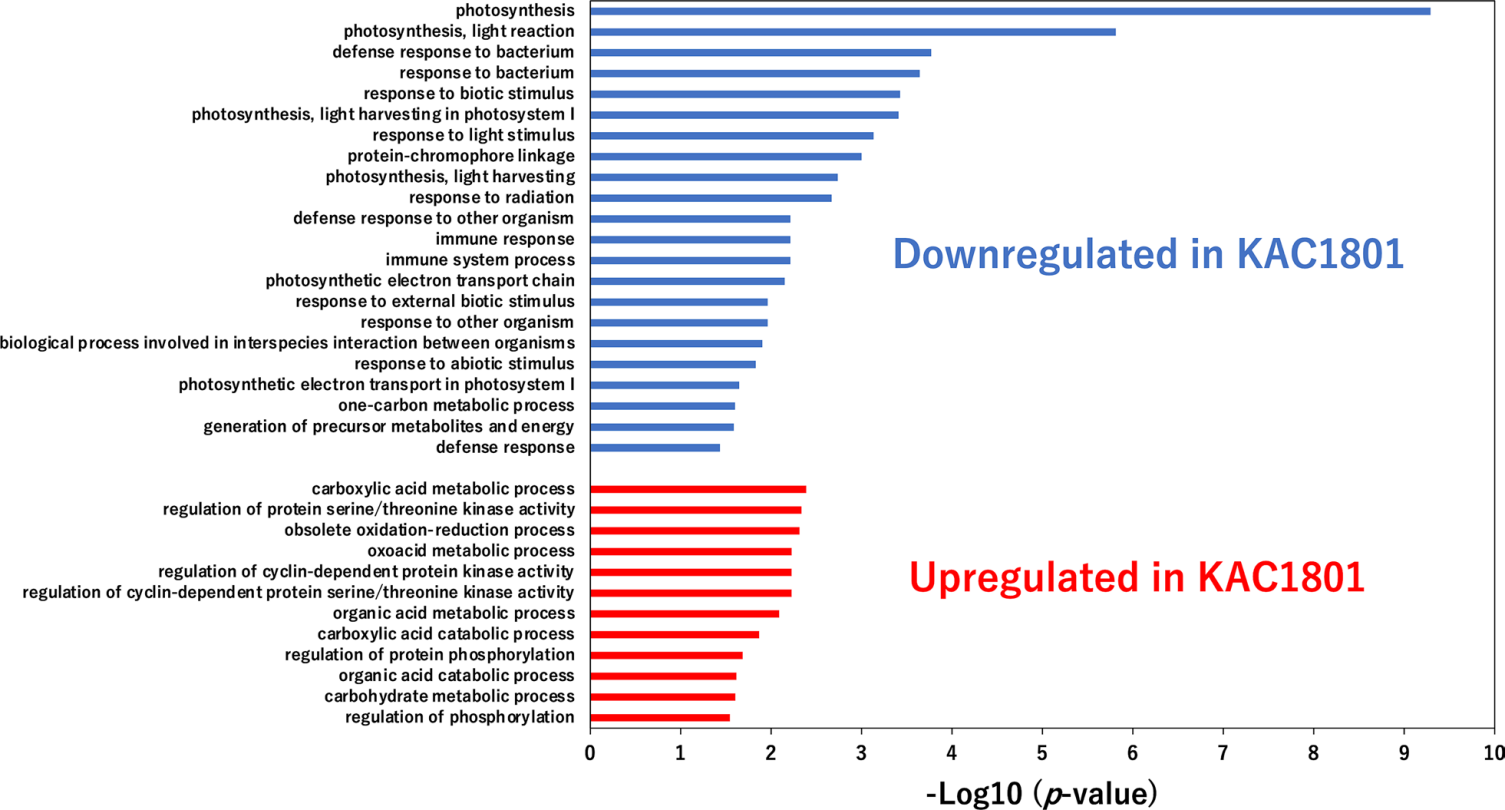
Gene ontology analysis of differentially expressed genes in *Chlamydomonas* sp. cells identified by RNA-seq analysis

To elucidate the underlying mechanism of the altered carbon distribution in KAC1801 at the transcript level, comprehensive gene expression profiling was performed via RNA-seq analysis using cells harvested at day 1.5. In total, 899 genes were identified as differently expressed genes (DEGs) between KOR1 and KAC1801. Among these, 275 and 624 genes were downregulated and upregulated in KAC1801, respectively (Additional file 2). Gene ontology (GO) analysis of the DEGs indicated that 22 and 12 categories were downregulated and upregulated in KAC1801, respectively (Fig. 6).

Among the downregulated categories, the expression of genes related to photosynthesis was reduced significantly in KAC1801 relative to KOR1. Among the downregulated genes in KAC1801, those associated with “photosynthesis” and “photosynthesis, light reaction” are listed in Table 2. These genes included chlorophyll *a*-*b* binding protein genes *LhcI-2*, *LhcI-3*, *LHCA2*, *LHCA9*, *LHCB4*, and *lhcb5*, which participate in light harvesting. This study also identified genes related to the Calvin cycle, including *SEBP1* (sedoheptulose-1,7-phosphatase) and *CHLRE_02g120150v5* (ribulose bisphosphate carboxylase/oxygenase small subunit) (Table 2). Genes related to stress responses, i.e., “defense response to bacterium,” “response to bacterium,” “response to biotic stimulus,” “response to radiation,” “defense response to other organism,” “immune response,” “immune system process,” “response to external biotic stimulus,” “response to other organism,” “biological process involved in interspecies interaction between organisms,” “response to abiotic stimulus,” and “defense response,” were also downregulated in KAC1801 (Fig. 6). Most of these were related to photosynthesis, including Rubisco activase (*Rca*), Rieske iron-sulfur subunit of the cytochrome b6f complex, chloroplast (*petC*), chloroplast ATP synthase delta chain (*ATPD*), peptidyl-prolyl cis–trans isomerase (*CYN38*), *LhcI-2*, *LhcI-3*, *LHCA2*, *LHCA9*, *LHCB4*, *lhcb5*, and *SEBP1* (Additional file 1: Table S2).

**Table 2:** Downregulated genes in KAC1801 associated with the “photosynthesis” and “photosynthesis, light reaction” categories

Protein ID (*Chlamydomonas reinhardtii*)	Gene IDs assigned by AUGUSTUS	Product	Gene name	log_2_FC	*p*-Value	FDR
PNW83466	g3062	Thylakoid membrane protein	-	–3.2	9.4 × 10^–6^	2.8 × 10^–4^
PNW86335	g958	PsaN	*psaN*	–2.6	6.5 × 10^–4^	8.8 × 10^–3^
PNW74812	g11087	Chlorophyll *a*-*b* binding protein, chloroplastic	*LHCA2*	–2.6	1.4 × 10^–4^	2.4 × 10^–3^
PNW76422	g4297	Chlorophyll *a*-*b* binding protein, chloroplastic	*LhcI-2*	–2.4	2.8 × 10^–4^	4.4 × 10^–3^
PNW87372	g10441	Ribulose bisphosphate carboxylase/oxygenase small chain	-	–2.4	7.5 × 10^–5^	1.5 × 10^–3^
PNW77185	g6306	Chlorophyll *a*-*b* binding protein, chloroplastic	*LhcI-3*	–2.2	5.0 × 10^–4^	7.1 × 10^–3^
PNW81164	g4727	Chlorophyll *a*-*b* binding protein, chloroplastic	*LHCA9*	–2.2	4.8 × 10^–4^	6.8 × 10^–3^
PNW70449	g84	Chlorophyll *a*-*b* binding protein, chloroplastic	*LHCB4*	–2.1	4.3 × 10^–4^	6.3 × 10^–3^
PNW84943	g6871	Photosystem I reaction center subunit VIII	*PSAI*	–2.0	5.7 × 10^–4^	7.9 × 10^–3^
PNW76554	g492	Rieske iron-sulfur subunit of the cytochrome b6f complex, chloroplast	*petC*	–2.0	9.1 × 10^–5^	1.7 × 10^–3^
PNW72305	g10904	Chlorophyll *a*-*b* binding protein, chloroplastic	*lhcb5*	–1.9	4.9 × 10^–4^	6.9 × 10^–3^
PNW79927	g6573	Oxygen evolving enhancer protein 3	*PSBQ*	–1.7	5.3 × 10^–4^	7.5 × 10^–3^
PNW76414	g6783	Chloroplast ATP synthase delta chain	*ATPD*	–1.6	6.8 × 10^–4^	9.1 × 10^–3^
PNW74805	g7396	OEE2-like protein of thylakoid lumen	*PSBP3*	–1.6	1.6 × 10^–4^	2.7 × 10^–3^
PNW85419	g2199	Sedoheptulose-1,7-bisphosphatase	*SEBP1*	–1.6	6.7 × 10^–4^	9.0 × 10^–3^
PNW71360	g3212	Cytochrome c6	*petJ*	–1.5	1.0 × 10^–4^	1.9 × 10^–3^

In contrast, the expression of genes related to carboxylic acid metabolism, especially those involved in the TCA cycle and its glyoxylate shunt, was upregulated in KAC1801 (Table 3). The glyoxylate shunt bypasses the two CO_2_-producing reactions in the TCA cycle, i.e., the conversion of isocitrate (Icit) to 2-OG and 2-OG to Suc [29, 30]. Upregulation of *CIS2* (citrate synthase), *ICL1* (isocitrate lyase), *MS1* (malate synthase), and *MDH2* (malate dehydrogenase) was observed in KAC1801 (Additional file 1: Fig. S4).

**Table 3:** Upregulated genes in KAC1801 of the “carboxylic acid metabolic process” category

Protein ID (*Chlamydomonas reinhardtii*)	Gene IDs assigned by AUGUSTUS	Product	Gene name	logFC	*p*-Value	FDR
PNW82533	g3710	Isocitrate lyase	*ICL1*	6.3	2.1 × 10^–9^	7.0 × 10^–7^
PNW84433	g6394	Malate synthase	*MS1*	5.2	2.6 × 10^–9^	8.1 × 10^–7^
PNW77089	g10773	Glycerol-3-phosphate dehydrogenase [NAD + dependent]	-	3.7	3.6 × 10^–8^	4.8 × 10^–6^
PNW71982	g10416	Acyl-coenzyme A oxidase	–	3.5	2.3 × 10^–8^	3.6 × 10^–6^
PNW77134	g2963	Threonine aldolase family protein	–	3.4	2.2 × 10^–7^	1.7 × 10^–5^
PNW78716	g5722	Aspartate aminotransferase	*AST1*	3.2	1.7 × 10^–8^	2.9 × 10^–6^
PNW85164	g2104	Cysteine dioxygenase	*CDO1*	2.8	6.2 × 10^–5^	1.3 × 10^–3^
PNW87457	g1547	*N*-Acetylglutamate synthase	*LCI8*	2.7	2.1 × 10^–6^	8.8 × 10^–5^
PNW76677	g6902	Acyl-coenzyme A oxidase	–	2.6	9.2 × 10^–6^	2.7 × 10^–4^
PNW71299	g11698	EF-hand domain-containing protein	–	2.4	7.8 × 10^–5^	1.5 × 10^–3^
PNW74464	g11056	Glyceraldehyde-3-phosphate dehydrogenase	*GAPC*	2.3	4.2 × 10^–5^	9.1 × 10^–4^
PNW70527	g9413	Acetyl-CoA acyltransferase	*ATO1*	2.0	7.0 × 10^–6^	2.2 × 10^–4^
PNW75961	g8276	Phosphofructokinase family protein	*PFK2*	1.9	2.4 × 10^–5^	5.9 × 10^–4^
PNW85759	g1229	Acetohydroxyacid dehydratase	*AAD1*	1.9	1.5 × 10^–5^	4.0 × 10^–4^
PNW82425	g3665	Arogenate/prephenate dehydrogenase	*AGD1*	1.9	6.6 × 10^–6^	2.1 × 10^–4^
PNW77127	g2953	Malate dehydrogenase	*MDH2*	1.9	3.5 × 10^–5^	7.8 × 10^–4^
PNW72803	g9028	SOR_SNZ domain-containing protein	–	1.8	1.5 × 10^–5^	3.9 × 10^–4^
PNW75447	g7819	6-Phosphogluconate dehydrogenase, decarboxylating	*gnd*	1.7	1.0 × 10^–5^	2.9 × 10^–4^
PNW85614	g2474	Malate dehydrogenase	*MDH2*	1.6	5.6 × 10^–5^	1.2 × 10^–3^
PNW75399	g7836	Cysteine desulfurase	*SUFS1*	1.5	3.1 × 10^–4^	4.8 × 10^–3^
PNW70105	g11069	Pseudouridine synthase domain-containing protein	–	1.7	1.4 × 10^–4^	2.4 × 10^–3^

## Discussion

In microalgae, nitrogen starvation not only is a strong trigger for lipid accumulation but also suppresses cell growth [17], which increases the risk of predation by environmental contaminants. Several microalgal strains have been developed to accumulate lipids under growth conditions [21–24]; however, a culture method to fully utilize the potential of this technology had not been established. *Chlamydomonas* sp. KAC1801 is a mutant which accumulates a high level of lipids under nitrate-replete conditions [24]. Using KAC1801, the present study achieved stable and continuous lipid production in a semi-continuous nitrate-replete culture system. KAC1801 produced 116.9 mg L^−1^ day^−1^ lipids (Table 4), which is comparable to the levels produced in previous semi-continuous and nitrogen-limited cultivation studies using microalgae strains that accumulated lipids under nitrogen starvation. For example, Han et al. and Hsieh and Wu achieved a lipid productivity of 115 and 139 mg L^−1^ day^−1^ in NaNO_3_- (~ 2.4 mM) and urea-limiting conditions (~ 0.5 mM), respectively [18, 19]. The present study developed a simple one-step semi-continuous cultivation method for biofuel production using a mutant strain that accumulated lipids under nitrate-replete conditions (> 11.8 mM NaNO_3_).

**Table 4:** Comparison of lipid productivity by semi-continuous and laboratory-scale cultivation

Strain	Nitrogen condition (concentration)	Lipid content (%)	Lipid productivity (mg L^−1^ day^−1^)	References
*Chlorella* sp.	Urea-limitation (0.5 mM)	38–47	139	Hsieh and Wu [19]
*Chlorella pyrenoidosa*	Nitrate-limitation (~ 2.4 mM)	20–30	115	Han et al. [18]
*Chlamydomonas* sp. KAC1801	Nitrate-replete (> 11.8 mM)	20–27	117	This study

In general, the photosynthetic pigment content as well as the ratio of nitrogen-containing compounds, such as proteins and chlorophylls, decrease under nitrogen-deficient conditions [31–34]. Under nitrogen-deficient conditions, chlorophylls and β-carotene decreased in *Chlamydomonas* sp. strains [25]. In the present study, both photosynthetic pigments (chlorophylls and β-carotene) and proteins decreased in KAC1801 compared to KOR1 (Fig. 2). Decreased protein content has been reported in a *Nannochloropsis* mutant grown under nutrient-replete conditions in which it can accumulate lipids [21]. Because nitrate consumption in KAC1801 was significantly lower than that in KOR1 (Fig. 1b), it was hypothesized that the intracellular level of nitrogen was decreased in KAC1801, which consequently induced nitrogen starvation-like responses, i.e., the accumulation of lipids and a decrease in photosynthetic pigments and proteins. The decrease in β-carotene content in KAC1801 may be due to lowered carbon flux for carotenoid synthesis, which was supported by the data of decreased pool sizes in the MEP pathway (Additional file 1: Fig. S3). The present study revealed that 2-OG and Suc decreased and Glu increased in KAC1801 (Fig. 4), suggesting increased GS activity. This may also be a part of the nitrogen starvation-like response because upregulation of GS and GOGAT genes under nitrogen-deficient conditions was reported in *Nannochloropsis* [35]. In KAC1801, the transcript levels of genes related to photosynthesis, for example, light harvesting (*LhcI-2*, *LhcI-3*, *LHCA2*, *LHCA9*, *LHCB4*, and *lhcb5*) and carbon fixation (*Rca* and *SEBP1*), were decreased (Fig. 6, Additional file 1: Table S2). These genes are known to be downregulated in *Chlamydomonas reinhardtii* and *Dunaliella tertiolecta* under nitrogen-deficient conditions [36, 37]. The *CYN38* gene, which contributes to the assembly and repair of photosystem II [38], was downregulated in KAC1801. This gene was also reported as a downregulated gene under nitrogen starvation conditions in *C. reinhardtii* [39]. In addition, pool size and turnover rate of metabolites in the Calvin cycle, including 3-PGA and S7P, were lower in KAC1801 (Figs. 4, 5). These results suggest that photosynthetic activity, especially light harvesting and carbon fixation, was lower in KAC1801, which may explain the reduced biomass production (Fig. 1a). KAC1801’s reduced photosynthetic activity may be due to decreased chlorophyll content (Fig. 2c), but this is uncertain because the mutant was created through random mutagenesis and thus may harbor mutations unrelated to pigment accumulation [24]. This study proposes that the nitrogen starvation-like response in KAC1801 was the cause of increased lipid accumulation under the nitrogen-replete conditions.

The transcript levels of genes involved in the TCA cycle and glyoxylate shunt (i.e., *ICL1*, *MS1*, *MDH2*, and *CIS2*) were higher in KAC1801 (Additional file 1: Fig. S4). This suggests enhancement of the glyoxylate cycle in KAC1801, which is advantageous for preventing emission of carbon sources because the CO_2_-producing reactions involved in the TCA cycle are bypassed by the glyoxylate shunt.

Lipid content was considerably higher in KAC1801 than in KOR1 (Fig. 1c), though the pool sizes and ^13^C fractions of the lipid precursors as well as the expression levels of genes related to lipid synthesis were similar or decreased between groups (Figs. 4, 5). Mutational analysis of KAC1801 was performed to identify genes responsible for the lipid accumulation, and mutations in 811 coding sequences were determined (data not shown). However, most of the identified genes were functionally uncharacterized, and no responsible gene was determined. Although further studies are required to elucidate the direct mechanism of lipid accumulation in KAC1801 under nitrate-replete conditions, it is hypothesized that KAC1801 may show enhanced lipid synthesis and decreased lipid degradation. For example, overexpression of the G3P acyltransferase *GPAT1* isoform in *Cyanidioschyzon merolae* increased lipid productivity by 56.1-fold without inhibiting growth [22]. In *C*. *reinhardtii*, genes encoding diacylglycerol acyltransferases (*DGAT1* and *DGAT2*), phospholipid:diacylglycerol acyltransferase (*PDAT*), and lysophosphatidic acid acyltransferase (*LPAAT*), which contribute to lipid synthesis, were upregulated under nitrogen-deficient conditions [40, 41]. In addition, knockout of the gene encoding phospholipase A_2_, which contributes to lipid degradation, improved lipid productivity in *C. reinhardtii* under growth conditions [42].

## Conclusions

This study describes a method for stable lipid production in the semi-continuous cultivation of *Chlamydomonas* sp. KAC1801 under nitrate-replete conditions by optimizing nitrate supply and cell density. KAC1801 constantly accumulated lipids at > 20% of DCW during 5 d of semi-continuous cultivation and achieved a lipid productivity of 117 mg L^−1^ day^−1^, which was comparable to previously reported levels of productivity under nitrogen-limiting conditions. Metabolome and transcriptome analyses revealed a nitrogen starvation-like response in KAC1801. Additionally, this one-step microalga lipid production method provides insights into the molecular responses associated with semi-continuous lipid production under nitrate-replete conditions.

## Methods

### Strains and culture conditions

Cultivation of microalgae, *Chlamydomonas* sp. KAC1801 [24] and the parental strain KOR1 [25], was performed using double-deck flasks and a BioTRON NC350 growth chamber (Nippon Medical & Chemical Instruments, Osaka, Japan) at 30 °C with shaking at 100 rpm. Continuous illumination with white fluorescent lamps was provided at 250 µmol photons m^−2^ s^−1^. The upper stage of the double-deck flask [24, 25] was supplemented with 70 mL of MB 12 N medium (18.7 mM NaNO_3_, 0.22 mM K_2_HPO_4_, 0.3 mM MgSO_4_·7H_2_O, 0.17 mM CaCl_2_·2H_2_O, 0.43 mM KH_2_PO_4_, and 0.43 mM NaCl) and trace elements as described in a previous report [43], including 2% (w/v) sea salt (Sigma-Aldrich, St. Louis, MO, USA). The CO_2_ concentration was adjusted to 2% by adding 50 mL of 2 M K_2_CO_3_/KHCO_3_ solution to the lower stage. After pre-cultivation for 5 days, the optical density at 750 nm (OD_750_) was measured using a UV mini-1240 UV–Vis spectrophotometer (Shimadzu, Kyoto, Japan). For semi-continuous cultivation, the cells were inoculated into new flasks every 24 h with an initial OD_750_ of 1.0.

### Measurement of biomass production

The culture broth was centrifuged at 8000×*g* for 1 min and washed once with ultrapure water. The cell pellet was lyophilized using an FDU-1200 (Tokyo Rikakikai, Tokyo, Japan). Daily biomass production (mg L^−1^) during semi-continuous cultivation was calculated as BC^x^ (mg L^−1^) − BC^y^ (mg L^−1^), where BC^x^ is the biomass concentration after 24 h of inoculation and BC^y^ is the biomass concentration after 0 h of inoculation.

### Measurement of nitrate concentration

The culture broth was centrifuged at 8000×*g* for 1 min. The absorbance of the supernatant was measured at 220 nm to determine the nitrate concentration using a calibration curve [44]. Daily nitrate consumption during semi-continuous cultivation was calculated using the following formula: Nitrate consumption (mM) = NC^x^ (mM) – NC^y^ (mM), where NC^x^ is the nitrate concentration after 0 h of inoculation, and NC^y^ is the nitrate concentration after 24 h of inoculation.

### Lipid analysis

Cells were harvested by centrifugation at 8000×*g* for 1 min, washed once with ultrapure water, and lyophilized. Lyophilized cells (2–3 mg) were suspended in 250 µL of methylation reagent A and 250 µL of methylation reagent B (Fatty Acid Methylation Kit, Nacalai Tesque, Kyoto, Japan), and fractured using 0.5 mm glass beads (YGB05) in a multi-bead shocker (MB1001C[S]; Yasui Kikai, Osaka, Japan) at 2700 rpm and 30 cycles of 1 min on and 1 min off at 4 °C. Lipids were extracted and esterified using the Fatty Acid Methylation Kit (Nacalai Tesque), according to the manufacturer’s instructions, and analyzed using a gas chromatograph-mass spectrometer (GC–MS)-QP2010 Plus (Shimadzu) equipped with a DB-23 capillary column (60 m, 0.25 mm internal diameter, 0.15 µm film thickness; Agilent Technologies, Santa Clara, CA, USA). Heptadecanoic acid (Sigma-Aldrich) was used as an internal standard for the quantification of fatty acids. The lipid content was calculated as the total intracellular fatty acid content per DCW [24, 25]. Daily lipid production (mg L^−1^) during semi-continuous cultivation was calculated as (BC^*x*^ [mg L^−1^] × LC^*x*^ [%])−(BC^*y*^ [mg L^−1^] × LC^*y*^ [%]), where BC^x^ is the biomass concentration after 24 h of inoculation, LC^*x*^ is the lipid content after 24 h of inoculation, BC^*y*^ is the biomass concentration after 0 h of inoculation, and LC^*y*^ is the lipid content after 0 h of inoculation.

### Carbohydrate analysis

Lyophilized cells (2–3 mg) were suspended in 2 mL of 4% (v/v) sulfuric acid and autoclaved at 120 °C for 30 min. The solution was neutralized by adding 1 mL of 22% (w/v) sodium carbonate. Cell debris was removed by centrifugation at 10,000×*g* for 10 min and subsequent filtration using a Shim-pack SPR-Pb column (Shimadzu). The glucose concentration was determined using a high-performance liquid chromatography (HPLC) system (Shimadzu) equipped with an Aminex HPX-87H column (9 μm, 300 mm × 7.8 mm; Bio-Rad Laboratories, Hercules, CA, USA). Soluble starch (CAS number: 9005-84-9, Nacalai Tesque) was used as the quantitative standard. The carbohydrate content was determined using a calibration curve [25].

### Protein analysis

Lyophilized cells (2–3 mg) were suspended in 0.2 mL of 1 N NaOH and incubated at 80 °C for 10 min. Subsequently, 1.8 mL of water was added and the solution was centrifuged at 12,000×*g* for 30 min. The protein concentration in the supernatant was analyzed using a Takara BCA protein assay kit (Takara Bio, Shiga, Japan) according to the manufacturer’s instructions [45, 46].

### Pigment analysis

Lyophilized cells (2–3 mg) were suspended in 500 μL of methanol:acetone (5:5 [v/v]) and fractured using 0.5 mm glass beads in a multi-bead shocker MB1001C(S) as described for lipid analysis. The samples were centrifuged at 10,000×*g* for 2 min at 4 °C, and the supernatant was transferred to a new microtube. The extraction procedure was repeated four times to obtain 2 mL of supernatant. The supernatant (330 μL) was dried in a vacuum using an evaporator CEV-3100 (EYELA, Tokyo, Japan), resuspended in 500 μL of chloroform:acetonitrile (2:8 [v/v]) containing 1 μM *trans*-β-apo-8-carotenal as an internal standard, and filtered using a 0.22 µm Cosmospin Filter G (Nacalai Tesque). Pigments were identified and quantified using an ACQUITY ultra liquid chromatography (UPLC) system equipped with a photodiode array detector and a BEH Shield RP18 column (1.7 μm, 2.1 mm × 100 mm; Waters, Milford, MA, USA) [25, 47].

### Metabolome analysis

Cells equivalent to 5 mg DCW were harvested using 10 µm pore size filters (Merck Millipore, Burlington, MA, USA), washed once with 20 mM ammonium carbonate, and immediately suspended in 1 mL of pre-cooled (–30 °C) methanol containing 36 µM piperazine-1,4-bis (2-ethanesulfonic acid) (Dojindo Laboratories, Kumamoto, Japan) and 36 µM l-methionine sulfone (Sigma-Aldrich) as internal standards. The suspension (500 µL) was subjected to cell disruption using 0.5 mm glass beads YGB05 in a multi-bead shocker MB1001C(S) as described for lipid analysis. Subsequently, 150 µL of chloroform and 50 µL of ultrapure water were added and mixed by vortexing for 10 s. After centrifugation at 14,000×*g* for 5 min at 4 °C, 400 µL of supernatant was collected, mixed with 200 µL of ultrapure water by vortexing for 10 s, and centrifuged at 14,000×*g* for 5 min at 4 °C. The upper phase was filtered using an Amicon Ultra-0.5 Centrifugal Filter Unit UFC5003BK (Merck Millipore) at 14,000×*g* for 50 min at 4 °C. The flow-through (300 μL) was dried in a vacuum using an evaporator CEV-3100 (EYELA). Dried samples were resuspended in 20 µL of ultrapure water and analyzed by CE-TOFMS using a G7100 CE and G6224AA liquid chromatograph/mass selective detector (LC/MSD) TOF system (Agilent Technologies) [25, 48].

### Dynamic metabolome analysis

To perform in vivo ^13^C labeling of newly synthesized metabolites using radiolabeled CO_2_, cells were harvested on day 1.5 of semi-continuous culture using 10 µm pore size filters (Merck Millipore) and resuspended in MB 12 N medium containing 2% (w/v) sea salt and 25 mM NaH^13^CO_3_ (Cambridge Isotope Laboratories, Tewksbury, MA, USA). After incubation under white fluorescent lamps at 250 μmol photons m^−2^ s^−1^ and shaking at 100 rpm, cells were harvested and the intracellular metabolites were analyzed as described for the metabolome analysis. The ^13^C labeling ratio was calculated as described in a previous report [25, 48].

### Genome analysis

The whole genome sequence of *Chlamydomonas* sp. was determined using the hybrid assembly method and Nanopore and Illumina KOR1 reads from a previous study [25]. Briefly, low-quality regions in the Nanopore long-reads were trimmed using Yanagiba v. 1.0.0 and assembled using Canu v. 1.7 [49]. Genome mapping analysis against the resulting assembly was performed using Burrows–Wheeler Aligner (BWA) v. 0.7.12 [50] and Illumina sequence reads; assembly polishing was performed using Pilon v. 1.23 against Illumina mapping data (https://github.com/broadinstitute/pilon). Prediction of gene coding sequences was performed using AUGUSTUS software v. 3.3.3 and a training set for *C. reinhardtii* (NCBI: txid3055) [51]. Functional assignments of the predicted genes were based on a BLASTP homology search using an E-value cutoff of 1e^−5^ against the previously reported *C. reinhardtii* genome [52]. The sequencing data obtained here were used as the reference in the RNA-seq analysis described below.

### Transcriptome analysis

Cells were harvested on day 1.5 of the semi-continuous culture by centrifugation at 12,000×*g* for 1 min, immediately frozen in liquid nitrogen, and stored at –80 °C. Total RNA was extracted using an RNeasy Plus Universal Kit (Qiagen, Tokyo, Japan), according to the manufacturer’s instructions. RNA integrity was determined using an Agilent Bioanalyzer 2100 and Agilent RNA 6000 Nano Kit (Agilent Technologies). Using a NEBNext Poly(A) mRNA Magnetic Isolation Module and NEBNext Ultra II RNA Library Prep Kit for Illumina (New England Biolabs, Ipswich, MA, USA), library preparation was performed using 500 ng of total RNA according to the manufacturer’s protocol, with 12 cycles of polymerase chain reaction (PCR). The library concentration and quality were assessed using an Agilent DNA 1000 Kit and the Agilent Bioanalyzer 2100 (Agilent Technologies). The library concentration was determined using a KAPA Library Quantification Kit (Kapa Biosystems, Wilmington, DE, USA) and confirmed using a StepOnePlus Real-Time PCR System (Thermo Fisher Scientific, Waltham, CA, USA). The cDNA library was sequenced using an Illumina NextSeq 500 platform, yielding 150 bp paired-end reads. Reads were generated in FASTQ format using conversion software bcl2fastq2 (Illumina, v. 2.18) and RNA-Seq analysis was performed using CLC Genomics Workbench v. 21.0.3 (Qiagen, Vedbæk, Denmark). Before mapping, adapter sequences were removed from the raw reads and low-quality bases from the start and end of single reads were clipped using a sliding window approach. Read mapping to the reference genome, read counts, and transcripts per million (TPM) were calculated for each gene using CLC Genomics Workbench v. 21.0.3 (Qiagen). Using edgeR [53], genes that showed a log_2_|FC|> 1 and false discovery rate (FDR) < 0.01 were identified as DEGs. Gene ontology analysis was performed using g:Profiler, a web server for functional enrichment analysis [54].

### Statistical analysis

The line and bar graphs presented in the figures represent the mean and standard deviation of the results of three replicate experiments. Statistical significance was determined using Welch’s *t*-test in R software (v. 3.3.3, R Foundation for Statistical Computing, Vienna, Austria).

## Supplementary Information

**Additional file 1: Fig. S1.** Biomass production, nitrate consumption, and lipid content in *Chlamydomonas* sp. during semi-continuous cultivation (*N* = 1). **Fig. S2.** Influence of inoculation cell density and nitrate concentration on lipid production of *Chlamydomonas* sp. **Fig. S3.** Pool size of metabolites in the carbohydrate synthesis and 2-*C*-methylerythritol 4-phosphate pathway (MEP pathway). **Fig. S4.** The upregulated genes in KAC1801 associated with the TCA cycle and glyoxylate shunt. **Table S1.** Influence of nitrate concentration and inoculation cell density during semi-continuous cultivation (N = 1). **Table S2.** All downregulated genes in KAC1801 included in the gene ontology of “defense response to bacterium,” “response to bacterium,” “response to biotic stimulus,” “response to radiation,” “defense response to other organism,” “immune response,” “immune system process,” “response to external biotic stimulus,” “response to other organism,” “biological process involved in interspecies interaction between organisms,” “response to abiotic stimulus,” and “defense response.”

**Additional file 2. **List of differently expressed genes (DEGs).

## Data Availability

The datasets used and/or analyzed in this study are available from the corresponding author on reasonable request. The sequence data used in this study have been deposited in the DNA Data Bank of Japan (DDBJ: https://www.ddbj.nig.ac.jp/index.html). The sequence data for assembling the KOR1 genome have been deposited with the DRA as accession number DRA011641 (Nanopore reads) and DRA013329 (Illumina reads). The contig data of KOR1 have been deposited with the DRA as accession numbers BQMZ01000001–BQMZ01000625. The RNA-seq sequence data for KOR1 and KAC1801 have been deposited with the DRA as accession number DRA013301.

## Abbreviations

2-OG: 2-Oxoglutarate
3-PGA: 3-Phosphoglycerate
AcCoA: Acetyl-CoA
ADP-glu: ADP-glucose
BWA: Burrows–Wheeler Aligner
CIS2: Citrate synthase 2
Cit: Citrate
CoA: Coenzyme A
DCW: Dry cell weight
DEGs: Differentially expressed genes
DGAT: Diacylglycerol acyltransferases
DXP: 1-Deoxy-d-xylulose 5-phosphate
E4P: Erythrose 4-phosphate
F6P: Fructose 6‐phosphate
FDR: False discovery rate
Fum: Fumarate
G3P: Glycerol 3-phosphate
G6P: Glucose 6‐phosphate
GC–MS: Gas chromatograph-mass spectrometer
Gln: Glutamine
GLO: Glyoxylate
Glu: Glutamate
GO: Gene ontology
GOGAT: Glutamate synthase
GS: Glutamine synthase
HPLC: High-performance liquid chromatography
Icit: Isocitrate
ICL1: Isocitrate lyase 1
LC/MSD: Liquid chromatograph/mass selective detector
LPAAT: Lysophosphatidic acid acyltransferase
Mal: Malate
MEcPP: 2-*C*-Methyl-d-erythritol-2,4-cyclopyrophosphate
MEP pathway: 2-*C*-Methylerythritol 4-phosphate pathway
MB: Modified Bold’s
MDH2: Malate dehydrogenase 2
MS1: Malate synthase 1
NiR: Nitrite reductase
NR: Nitrate reductase
OAA: Oxaloacetate
OD: Optical density
PCR: Polymerase chain reaction
PDAT: Phospholipid:diacylglycerol acyltransferase
PEP: Phosphoenolpyruvate
Pyr: Pyruvate
R5P: Ribose 5-phosphate
S7P: Sedoheptulose 7-phosphate
SEBP1: Sedoheptulose-1,7-phosphatase
Suc: Succinate
SucCoA: Succinyl-CoA
TCA cycle: Tricarboxylic acid cycle
TPM: Transcripts per million
UPLC: Ultra-performance liquid chromatography
