# Supplementary material for: Development of a stable semi-continuous lipid production system of an oleaginous Chlamydomonas sp. mutant using multi-omics profiling
Source: Biotechnol Biofuels Bioprod. 2022 Sep 16;15:95. doi: 10.1186/s13068-022-02196-w (PMC9482161; doi:10.1186/s13068-022-02196-w)
Supplement: Supplementary file 1 — Additional file 1: Fig. S1. Biomass production, nitrate consumption, and lipid content in Chlamydomonas sp. during semi-continuous cultivation (N = 1). Fig. S2. Influence of inoculation cell density and nitrate concentration on lipid production of Chlamydomonas sp. Fig. S3. Pool size of metabolites in the carbohydrate synthesis and 2-C-methylerythritol 4-phosphate pathway (MEP pathway). Fig. S4. The upregulated genes in KAC1801 associated with the TCA cycle and glyoxylate shunt. Table S1. Influence of nitrate concentration and inoculation cell density during semi-continuous cultivation (N = 1). Table S2. All downregulated genes in KAC1801 included in the gene ontology of “defense response to bacterium,” “response to bacterium,” “response to biotic stimulus,” “response to radiation,” “defense response to other organism,” “immune response,” “immune system process,” “response to external biotic stimulus,” “response to other organism,” “biological process involved in interspecies interaction between organisms,” “response to abiotic stimulus,” and “defense response.” [file 13068_2022_2196_MOESM1_ESM.docx]

**Development of a stable semi-continuous lipid production system of an oleaginous *Chlamydomonas* sp. mutant using multi-omics profiling**

Tomoki Oyama^1^, Yuichi Kato^2^, Ryota Hidese^1,2^, Mami Matsuda^1^, Minenosuke Matsutani^3^, Satoru Watanabe^4^, Akihiko Kondo^1,2,5^, Tomohisa Hasunuma^1,2*^

^1^Graduate School of Science, Technology and Innovation, Kobe University, 1-1 Rokkodai, Nada, Kobe 657-8501, Japan.

^2^Engineering Biology Research Center, Kobe University, 1-1 Rokkodai, Nada, Kobe 657-8501, Japan.

^3^NODAI Genome Research Center, Tokyo University of Agriculture, 1-1-1 Sakuragaoka, Setagaya, Tokyo 156-8502, Japan.

^4^Department of Bioscience, Tokyo University of Agriculture, 1-1-1 Sakuragaoka, Setagaya, Tokyo 156-8502, Japan.

^5^Department of Chemical Science and Engineering, Graduate School of Engineering, Kobe University, 1-1 Rokkodai, Nada, Kobe 657-8501, Japan.


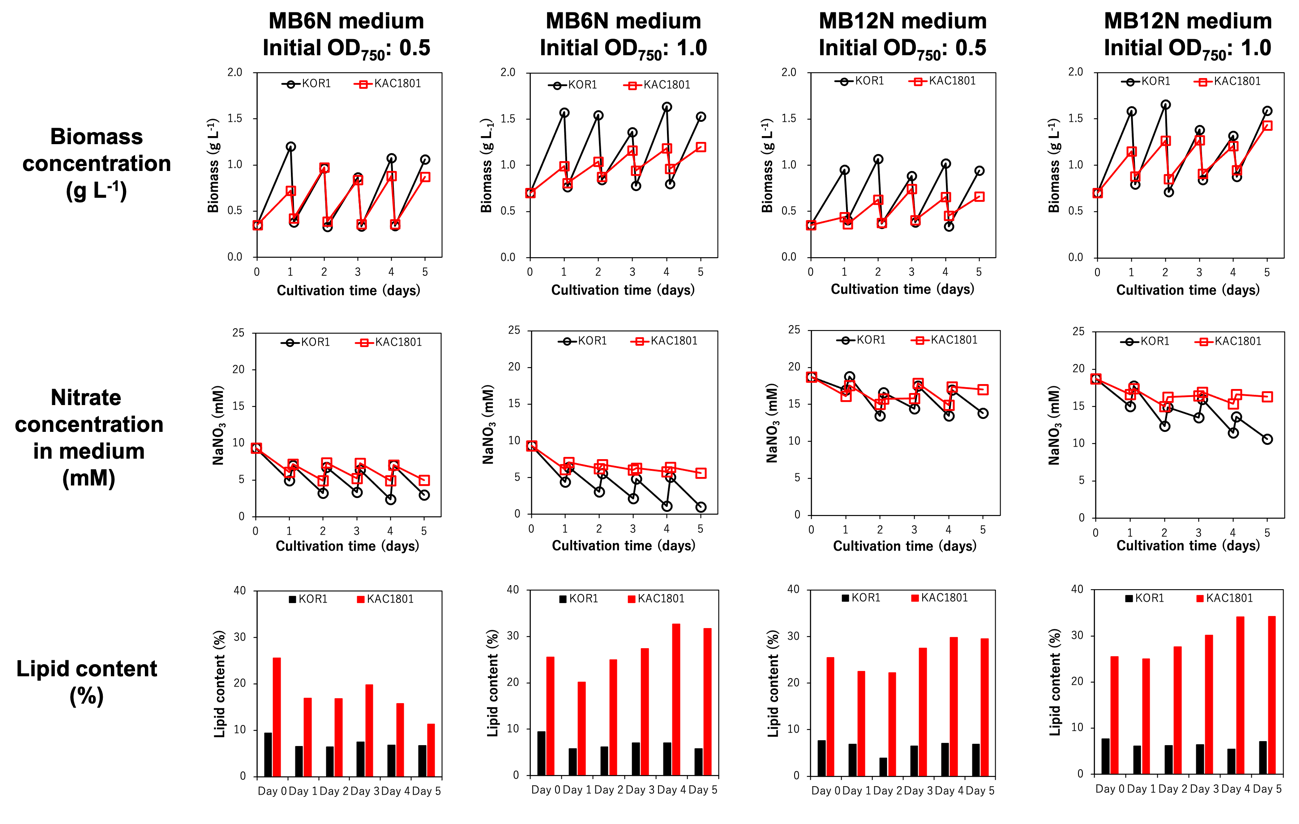


**Fig. S1.** Biomass production, nitrate consumption, and lipid content in *Chlamydomonas* sp. during semi-continuous cultivation (N = 1).

Microalgae were cultured using MB medium with 2% (*w*/*v*) sea salt, containing either 9.3 mM NaNO_3_ (6N) or 18.7 mM NaNO_3_ (12N), under continuous illumination at 250 µmol photons m^-2^ s^-1^ with white fluorescence lamps, and supplemented with 2% CO_2_ at 30 °C on a rotary shaker at 100 rpm. For semi-continuous cultivation, cells were inoculated every 24 h at an initial cell density of OD_750_ = 0.5 or 1.0.


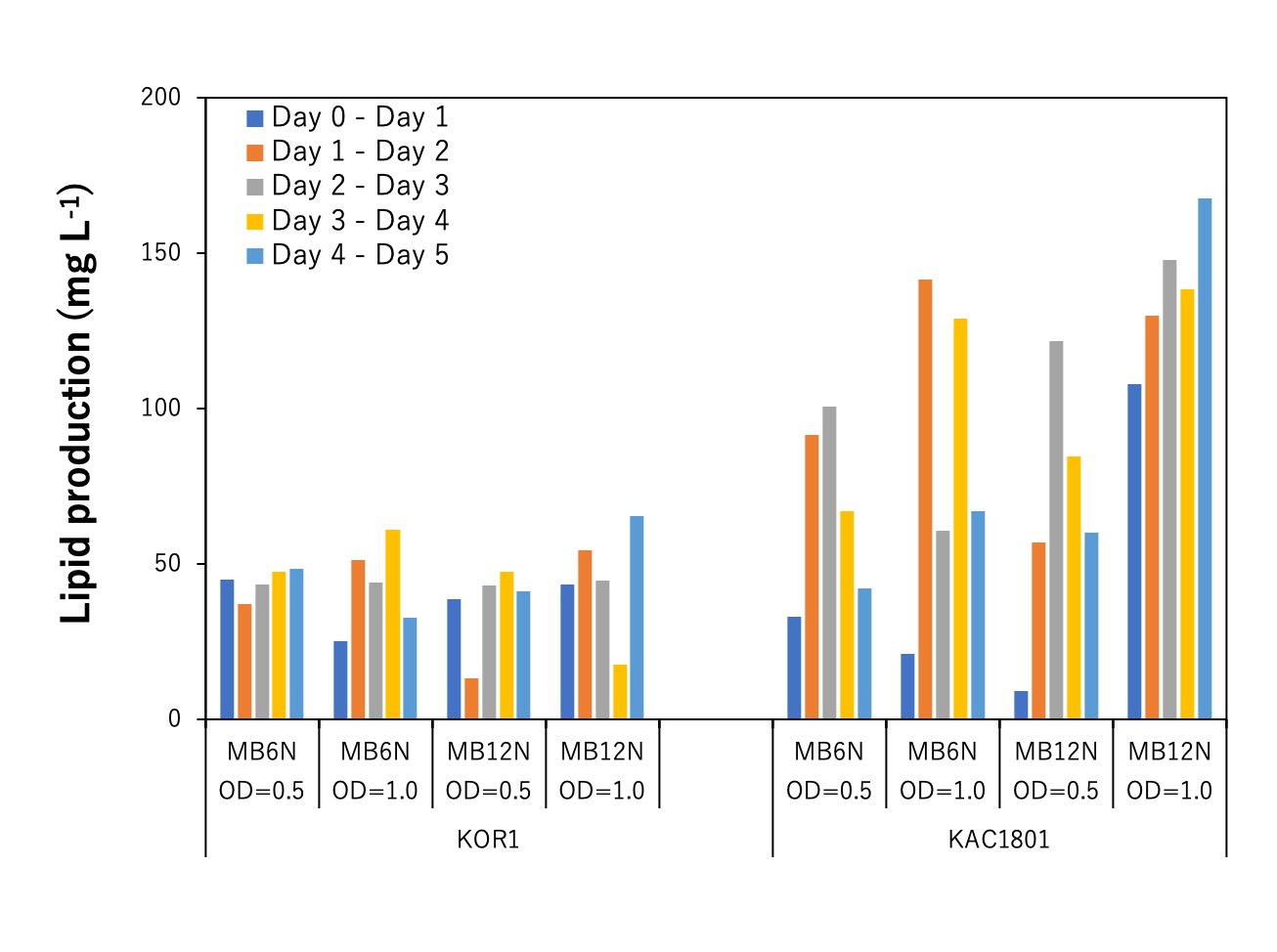


**Fig. S2.** Influence of inoculation cell density and nitrate concentration on lipid production in *Chlamydomonas* sp.


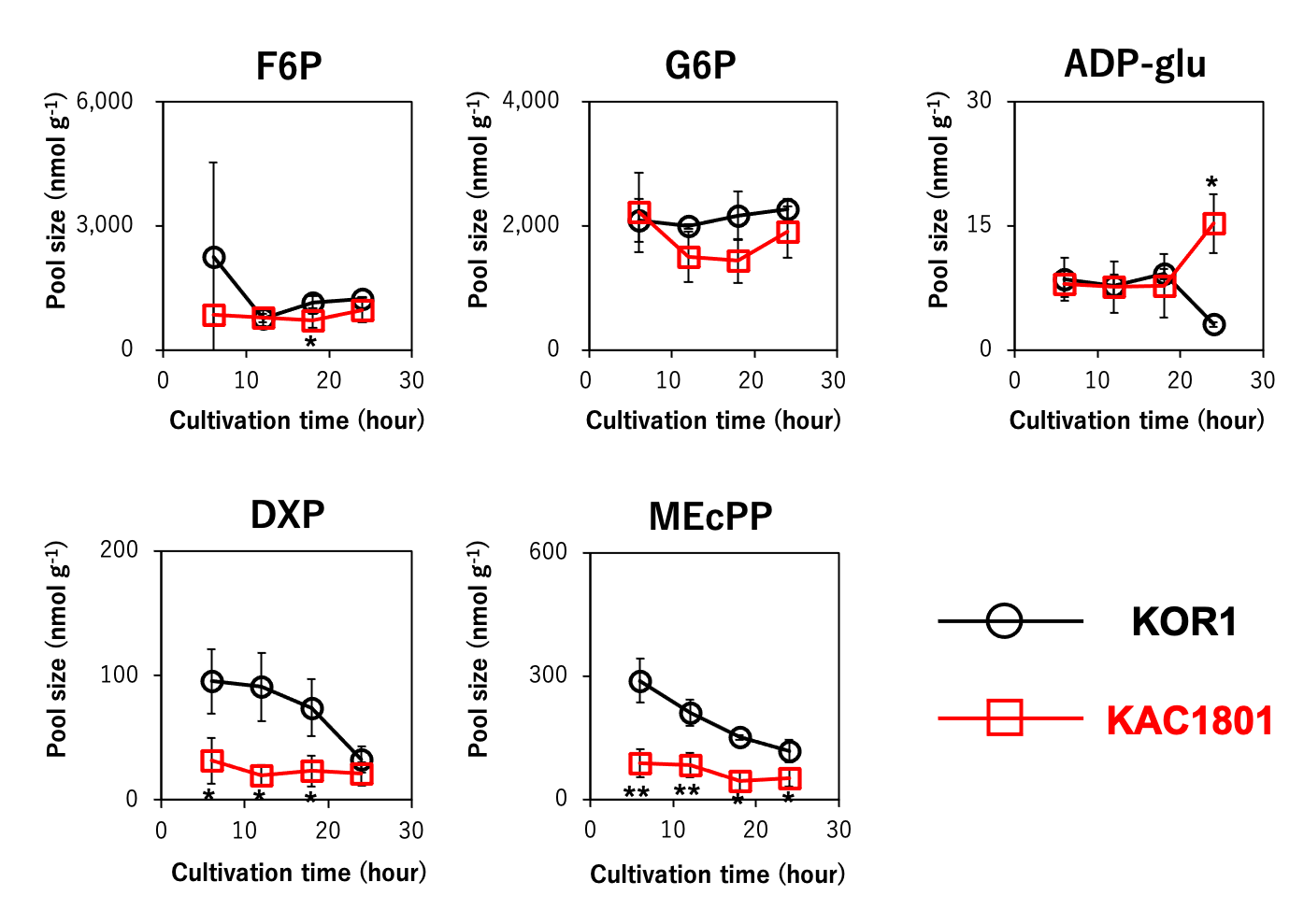


**Fig. S3.** Pool size of metabolites in the carbohydrate synthesis and 2-*C*-methylerythritol 4-phosphate (MEP) pathways.

KOR1 and KAC1801 cells from days 1 to 2 in the semi-continuous culture were subjected to metabolome analysis. Error bars indicate the standard deviation of three replicate experiments (*: *p* < 0.05, **: *p* < 0.01 via Welch’s *t*-test).

F6P, fructose 6‐phosphate; G6P, glucose 6‐phosphate; ADP-glu, ADP-glucose; DXP, 1-deoxy-d-xylulose 5-phosphate; MEcPP, 2-*C*-methyl-d-erythritol-2,4-cyclopyrophosphate.


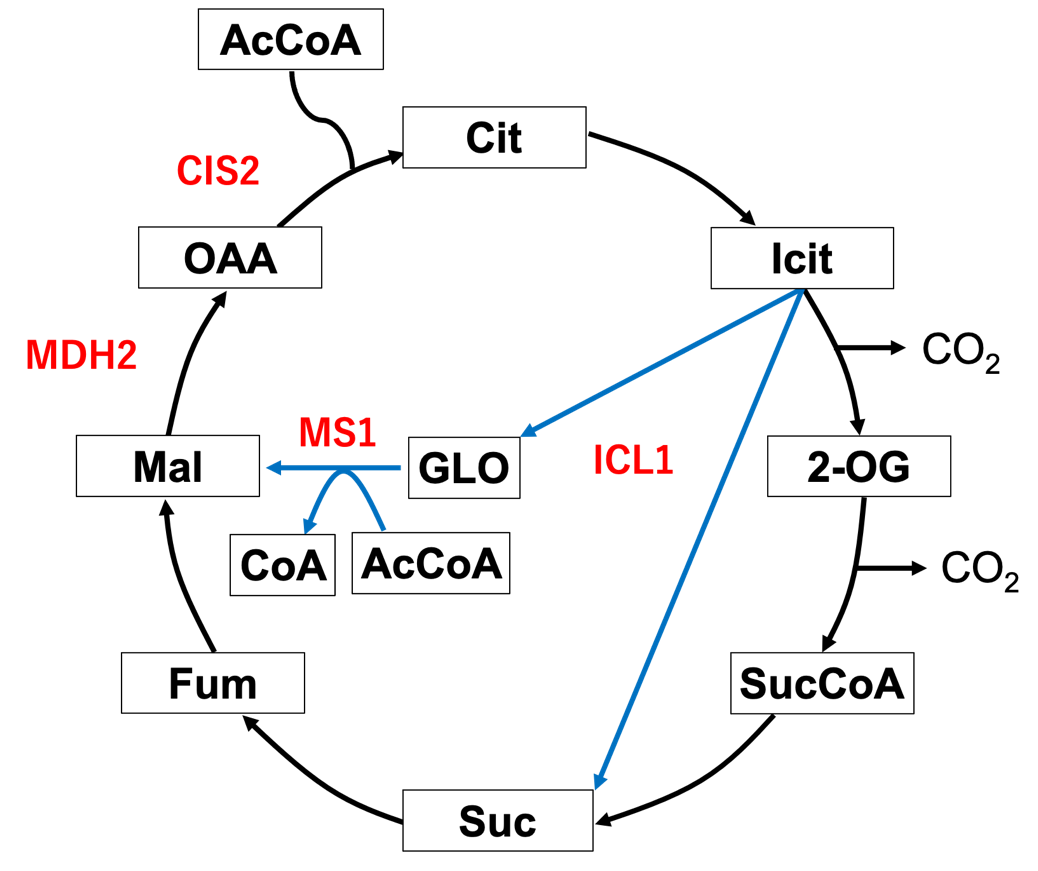


**Fig. S4.** The upregulated genes in KAC1801 associated with the TCA cycle and its glyoxylate shunt.

Blue arrows indicate reactions involved in the glyoxylate shunt. Genes upregulated in KAC1801 are presented in red.

Cit, citrate; Icit, isocitrate; 2-OG, 2-oxoglutarate; GLO, glyoxylate; AcCoA, acetyl-CoA; CoA, coenzyme A; SucCoA, succinyl-CoA; Suc, succinate; Fum, fumarate; Mal, malate; OAA, oxaloacetate.

ICL1, isocitrate lyase; MS1, malate synthase; MDH2, malate dehydrogenase; CIS2, citrate synthase.

**Table S1**. Influence of nitrate concentration and inoculation cell density during semi-continuous cultivation (N = 1).

| Strain | Medium | Nitrate concentration (mM) | Inoculation cell density | Biomass production  (mg L^–1^) | Lipid content  (%) | Lipid production  (mg L^–1^) |
| --- | --- | --- | --- | --- | --- | --- |
| KOR1 | MB6N including 2% sea salt | 9.3 | OD_750_ = 0.5 | 687.8 | 7.2 | 44.4 |
|  |  |  | OD_750_ = 1.0 | 756.3 | 6.9 | 42.9 |
|  | MB12N including 2% sea salt | 18.7 | OD_750_ = 0.5 | 606.0 | 6.5 | 36.9 |
|  |  |  | OD_750_ = 1.0 | 725.0 | 6.5 | 45.3 |
| KAC1801 | MB6N including 2% sea salt | 9.3 | OD_750_ = 0.5 | 480.7 | 17.7 | 67.0 |
|  |  |  | OD_750_ = 1.0 | 263.4 | 27.1 | 84.0 |
|  | MB12N including 2% sea salt | 18.7 | OD_750_ = 0.5 | 236.1 | 26.2 | 66.6 |
|  |  |  | OD_750_ = 1.0 | 407.9 | 29.4 | 138.4 |

The values of biomass production, lipid content, and lipid production are the averages of 5 d cultivations.

Table S2. All downregulated genes in KAC1801 included in the gene ontology of “defense response to bacterium”, “response to bacterium”, “response to biotic stimulus”, “response to radiation”, “defense response to other organism”, “immune response”, “immune system process”, “response to external biotic stimulus”, “response to other organism”, “biological process involved in interspecies interaction between organisms”, “response to abiotic stimulus” and “defense response”.

| Protein ID  (*Chlamydomonas reinhardtii*) | Gene IDs assigned by AUGUSTUS | Product | Gene name | Log_2_FC | *p*-Value | FDR |
| --- | --- | --- | --- | --- | --- | --- |
| PNW84312 | g5506 | Rubisco activase | *Rca* | -3.5 | 3.6×10^-6^ | 1.3×10^-4^ |
| PNW76554 | g492 | Rieske iron-sulfur subunit of the cytochrome b6f complex, chloroplast | *petC* | –2.0 | 9.1×10^-5^ | 1.7×10^-3^ |
| PNW85507 | g2317 | Peptidyl-prolyl cis-trans isomerase, cyclophilin-type | *CYN38* | -1.9 | 4.7×10^-4^ | 6.7×10^-3^ |
| PNW77146 | g6279 | Inorganic pyrophosphatase | *ppa1* | -1.9 | 1.1×10^-4^ | 2.0×10^-3^ |
| PNW76414 | g6783 | Chloroplast ATP synthase delta chain | *ATPD* | -1.6 | 6.8×10^-4^ | 9.1×10^-3^ |
| PNW85419 | g2199 | Sedoheptulose-1,7-bisphosphatase | *SEBP1* | -1.6 | 6.7×10^-4^ | 9.0×10^-3^ |
| PNW82314 | g9690 | SCP domain-containing protein | - | -4.2 | 8.9×10^-7^ | 4.6×10^-5^ |
| PNW88037 | g4289 | Predicted protein | - | -4.3 | 1.3×10^-5^ | 3.4×10^-4^ |
| PNW83466 | g3062 | Thylakoid membrane protein | - | -3.2 | 9.4×10^-6^ | 2.8×10^-4^ |
| PNW74812 | g11087 | Chlorophyll a-b binding protein, chloroplastic | *LHCA2* | -2.6 | 1.4×10^-4^ | 2.4×10^-3^ |
| PNW76422 | g4297 | Chlorophyll a-b binding protein, chloroplastic | *LhcI-2* | -2.4 | 2.8×10^-4^ | 4.4×10^-3^ |
| PNW77185 | g6306 | Chlorophyll a-b binding protein, chloroplastic | *LhcI-3* | -2.2 | 5.0×10^-4^ | 7.1×10^-3^ |
| PNW81164 | g4727 | Chlorophyll a-b binding protein, chloroplastic | *LHCA9* | -2.2 | 4.8×10^-4^ | 6.8×10^-3^ |
| PNW70449 | g84 | Chlorophyll a-b binding protein, chloroplastic | *LHCB4* | -2.1 | 4.3×10^-4^ | 6.3×10^-3^ |
| PNW72305 | g10904 | Chlorophyll a-b binding protein, chloroplastic | *lhcb5* | -1.9 | 4.9×10^-4^ | 6.9×10^-3^ |
